# Defects in muscarinic receptor-coupled signal transduction in isolated parotid gland cells after *in vivo* irradiation: evidence for a non-DNA target of radiation

**DOI:** 10.1038/sj.bjc.6602365

**Published:** 2005-01-25

**Authors:** R P Coppes, A Meter, S P Latumalea, A F Roffel, H H Kampinga

**Affiliations:** 1 Department of Radiation and Stress Cell Biology, University of Groningen, Ant. Deusinglaan 1, 9713 AV Groningen, The Netherlands; 2 Department of Molecular Pharmacology, University of Groningen, Ant. Deusinglaan 1, 9713 AV Groningen, The Netherlands; 3 Department of Radiation Oncology, University of Groningen, Ant. Deusinglaan 1, 9713 AV Groningen, The Netherlands

**Keywords:** head and neck cancer, irradiation, parotid gland, intracellular signalling

## Abstract

Radiation-induced dysfunction of normal tissue, an unwanted side effect of radiotherapeutic treatment of cancer, is usually considered to be caused by impaired loss of cell renewal due to sterilisation of stem cells. This implies that the onset of normal tissue damage is usually determined by tissue turnover rate. Salivary glands are a clear exception to this rule: they have slow turnover rates (>60 days), yet develop radiation-induced dysfunction within hours to days. We showed that this could not be explained by a hypersensitivity to radiation-induced apoptosis or necrosis of the differentiated cells. In fact, salivary cells are still capable of amylase secretion shortly after irradiation while at the same time water secretion seems specifically and severely impaired. Here, we demonstrate that salivary gland cells isolated after *in vivo* irradiation are impaired in their ability to mobilise calcium from intracellular stores (Ca^2+^_i_), the driving force for water secretion, after exposure to muscarinic acetylcholine receptor agonists. Using radioligand-receptor-binding assays it is shown that radiation caused no changes in receptor density, receptor affinity nor in receptor-G-protein coupling. However, muscarinic acetylcholine agonist-induced activation of protein kinase C alpha (PKC*α*), measured as translocation to the plasma membrane, was severely affected in irradiated cells. Also, the phorbol ester PMA could no longer induce PKC*α* translocation in irradiated cells. Our data hence indicate that irradiation specifically interferes with PKC*α* association with membranes, leading to impairment of intracellular signalling. To the best of our knowledge, these data for the first time suggest that, the cells' capacity to respond to a receptor agonist is impaired after irradiation.

During the radiotherapeutic treatment of cancer, the normal tissue toxicity limits the treatment dose. In general, high sensitivity of tissues to radiation-induced damage is associated with rapid cell turnover and high proliferation activity. For example, bone marrow and intestine are highly radiosensitive, whereas the central nervous system is relatively radioresistant. The salivary gland forms an exception to this rule. In spite of its relatively low cycling activity and highly differentiated character, the function of the salivary gland is severely compromised within a few days after relatively low doses of ionising radiation (Coppes *et al*, 1997a, 1997b; Taylor and Miller, 1999; Burlage *et al*, 2001). Classical clonogenic death cannot explain this loss in function as the fraction of proliferating cells is usually below 1% (Peter *et al*, 1994). Furthermore, lowering the dose rate enhanced rather than reduced the acute drop in gland function (Vissink *et al*, 1992), suggesting that classical radiobiological parameters of DNA-damage-dependent cellular recovery do not apply. As an alternative hypothesis, it has been suggested that interphase acinar cells are highly susceptible to radiation-induced apoptosis (Stephens *et al*, 1986). In rats, however, a 50% drop in secretory capability 3 days after irradiation was associated with less than 3% of apoptosis activity (Paardekooper *et al*, 1998). Moreover, accurate cell counting demonstrated no significant cell loss within 10 days after irradiation (Coppes *et al*, 2000), implying that disappearance by cell death pathways other than classical apoptotic routes also cannot explain the acute loss in function of the salivary glands after radiation. Thus, loss in gland function must be due to radiation-induced cellular dysfunction rather than cell loss and/or impaired cell renewal.

The parotid gland consists of ducts and grape-like acini containing acinar cells responsible for the secretion of primary saliva. The watery secretion by the acinar cells in the salivary gland is predominantly mediated via the parasympathetic nervous system, releasing acetylcholine, which subsequently stimulates G-protein-coupled, muscarinic acetylcholine receptors on the acinar cells (Nauntofte, 1992). When stimulated, these receptors, which are of 93% of the M3 subtype (Dai *et al*, 1991), induce Gq-protein-coupled activation of phospholipase C (PLC), which hydrolyses phosphatidyl-inositol-4,5-bisphosphate (PIP_2_). This leads to the formation of inositol-1,4,5-trisphosphate (IP_3_), which mobilises Ca^2+^ from intracellular stores, and diacylglycerol (DAG), which activates protein kinase C (PKC) (Nishizuka, 1992; Baum *et al*, 1993, Sawali *et al*, 1993). An increase in intracellular free Ca^2+^ (Ca^2+^_i_) concentration is the predominant mechanism triggering the fluid secretion from acinar cells (Melvin, 1999), whereas PKC activates the secretion of low amounts of proteins in the saliva (Tojyo *et al*, 1992). Changes herein may, however, be difficult to show *in vivo* due to the relatively low amount induced through simulation of this pathway (Coppes *et al*, 2000). The bulk of protein secretion is mediated through stimulation of *β*-adrenoceptors and subsequent cAMP-induced exocytosis (Baum *et al*, 1993). Interestingly, whereas fluid secretion is impaired early after radiation, protein secretion of the parotid gland is not (Bodner *et al*, 1984, Coppes *et al*, 2001) or of the submandbular gland is less (Takagi *et al*, 2003) affected. This observation not only confirms that cell loss cannot be the cause of impaired gland function but also points to the possibility that radiation may affect specific aspects of the intracellular receptor-mediated signalling in acinar cells. Indirect evidence from radioprotection experiments supports this hypothesis. In these experiments, radioprotective actions of *α*-adrenergic or muscarinic acetylcholine receptor agonists were annihilated by simultaneous stimulation of crossinhibitory *β*-adrenergic receptor signalling pathways (Coppes *et al*, 2001).

To directly determine whether radiation indeed affects receptor-effector signalling, rat parotid glands were locally irradiated *in vivo*, which induces a significant reduction in salivary secretion independent from other factors than damage to the glands (Coppes *et al*, 1997a, 1997b; and Takeda *et al*, 2003). After killing the animal, acinar cells were isolated to investigate (1) their capability to mobilise calcium upon receptor stimulation, (2) their muscarinic acetylcholine receptor numbers, (3) the muscarinic acetylcholine receptor coupling to G-proteins and (4) translocation of PKC*α*. Our combined data demonstrate that radiation interferes with agonist-stimulated muscarinic acetylcholine receptor-mediated signalling pathways in parotid gland cells. This consequently may be the cause of the impaired cell and tissue function. This would point to a novel, radiobiological mechanism of normal tissue damage after *in vivo* irradiation.

## MATERIALS AND METHODS

### Animals

Young adult (8–9 week old) male albino Wistar rats (strain Hds/Cpb: WU), weighing approximately 230 g, purchased from Harlan CPB Rijswijk, the Netherlands, were used in all studies. They were kept in polycarbonate cages (six rats per cage) under a 14 : 10-h light : dark cycle. The rats were housed for 10 days prior irradiation. Food (RMH-B, Hope Farms, Woerden, the Netherlands) and water was given *ad libitum*. All experiments were performed in agreement with the Netherlands Experiments on Animal Act (1977), the European Convention for the Protection of Vertebrates Used for Experimental Purposes (Strasbourg, 18.III.1986) and met the standards required by the UKCCCR Guidelines (UKCCCR, 1998).

### Parotid gland irradiation

The rats were irradiated as previously described (Coppes *et al*, 1997a). Prior to irradiation all rats were anaesthetised by an i.p. injection of Ketamine (Ketalar, 60 mg kg^−1^) and Xylazine (Rompun, 2.5 mg kg^−1^). A 6-mm lead shield with a portal of 2 × 5 cm was positioned over the body of the rat so that, except for the parotid/submandibular region, the body, including the oral cavity, was excluded from the irradiation field. Both glands were irradiated with a single dose of 15 Gy. The X-ray apparatus (Mueller MG 300, Philips, Eindhoven, The Netherlands) was operated at 15 mA, 200 kV (filters : 0.5 mm copper and 0.5 mm aluminium; HVL=1 mm Cu). The treatment distance to the focal spot of the skin was 32.5 cm, leading to a dose rate at the gland level of 1.0 Gy min^−1^. This dose rate was determined in air with a calibrated electrometer and ionisation chamber combination (Keithley 35040+NE-2571). The tissue outside the primary beam received less than 1% of the dose applied.

### Preparation of parotid acinar cells

The isolation was based on a protocol developed for acinar cells of the parotid gland (Oliver, 1980). At the appropriate time after irradiation, the rats were anaesthetised using 60 mg kg^−1^ Brietal i.p. Both parotid glands were removed and were cut into small pieces and put in a spinner flask containing 25 ml RPMI+ 5 mM HEPES+60 *μ*l 0.5 M EGTA. After 5 min incubation at 35°C, the solution was decanted and the tissue was again incubated for 30 min in 15 ml RPMI with 5 mM HEPES containing 1 ml collagenase and 0.5 ml hyaluronidase (8100 U/dissolved in 0.5 ml HANK's w/o Ca and Mg). Enzymatic digestion was stopped using 10 ml RPMI containing 4% BSA. Hereafter, the cell suspension was passed through a nylon gauze (100 *μ*m). The filtrate was centrifuged for 5 min at 1500 r.p.m. and the pellet was dissolved in RPMI+4% BSA with an end concentration of 1 × 10^6^ cells ml^−1^. Viability was assessed by 0.1% trypan blue exclusion. A maximum of three cells per clump was accepted. The tissue/cells were gas dispersed (5% O_2_) until measurements.

### Measurements of free Ca^2+^ concentrations

[Ca^2+^]_i_ was determined using Ca^2+^-sensitive fluorescent indicator as described by Dissing *et al* (1990). Parotid acinar cell suspension (2 ml) was incubated for 30 min at 37°C with 4 *μ*l of 0.5 mM Fura-2 A/M. The loaded cell suspension was centrifuged and washed twice with KREBS buffer (142 mM NaCl, 5 mM KCl, 1 mM MgCl_2_, 20 mM NaHCO_3_, 4 mM Na_2_HPO_4_, 4 mM NaH_2_PO_4_, pH=7.25) to remove extracellular Fura-2 A/M. After washing the samples, 1.5 ml Fura-2 loaded suspension was put in a 2 ml quarts cuvet. Then, 1.5 *μ*l 1 M CaCl_2_ was added to yield 1 mM extracellular CaCl_2_, to be able to measure intra- and extracellular Ca responses. The cells were stimulated with methacholine in concentrations ranging from 10 *μ*M to 1 mM. [Ca^2+^]_i_ was calculated from the measurements of the ratio of fluorescence intensities obtained at 340 and 380 nm. The measurements of [Ca^2+^]_i_ in acinar suspensions were performed with a Hitachi F-4000 fluorescence spectrophotometer with excitation wavelengths of 340 and 380 nm and a emission wavelength of 505 nm. Calibration of [Ca^2+^]_i_ was performed for each measurement by adding an excess of Triton X 100/EDTA (*F*_min_), CaCl_2_ (*F*_max_) and MnCL_2_ (*F*_auto_), respectively. Fluorescence ratios of 340/380 nm excitation and 505 nm emission were converted to [Ca^2+^]_i_ according to Grynkiewicz *et al* (1985).

### Measurements of receptor number and coupling to G-protiens

#### Membrane preparation

At the appropriate time after irradiation, the rats were anaesthetised using 60 mg kg^−1^ Brietal i.p. Both parotid glands were removed and were rapidly transferred into ice-cold homogenisation buffer (20 mM Tris-HCl pH=7.4, 1 mM EDTA, 0.1 mM PMSF). The tissue was washed and cut into small pieces and diluted in 10-fold homogenisation buffer. Homogenisation was performed using a polytron (PT10-35 with PTA probe) three times for 10 s, whereafter the suspension was filtered through a two layer gauze at 4°C. The homogenate was centrifuged for 20 min at 5000 × **g** and the supernatant was recentrifuged for 60 min at 70 000 × **g**. The resulting pellet was resuspended in the homogenisation buffer with a dilution of 1 : 80 and kept at −80°C.

The membrane protein concentration was determined by the method of Lowry *et al* (1951) with BSA as the standard.

#### Radioligand binding assay

To determine the maximal binding (*B*_max_) and the dissociation constant of the hot ligand (^3^H-NMS, Amersham, Life Science, 81 Ci mmol^−1^, 1 mCi ml^−1^), a saturation curve was made. For this curve, 400 *μ*l membrane suspension (1 mg ml^−1^) was incubated with a concentration range of the hot ligand. After 30 min, incubation at 37°C, the reactions were terminated by adding ice-cold 50 mM NaPO_4_ buffer followed by filtration through Whatmann GF/B filters. These filters were rinsed twice with 4 ml ice-cold buffer. The filters were put in polyethylene counting vials with 3 ml counting solution (Aqualuma Plus^R^). These vials were shaken for 2 h and counted in a Packard Tri-Carb 4450 liquid scintillation counter. To measure the dissociation constant of methacholine, with and without the GTP-analogue 5′-guanylylimido diphosphate (GppNHp), a competition curve was created. In this case, the concentration of hot ligand was constant and different concentrations of methacholine were added to the membrane suspension. Incubation and quantitation of the radioactivity was performed as described above.

Nonspecific binding was determined as the amount of ^3^H-NMS bound in the presence of excess/1 *μ*M dexetimide. Binding parameters were calculated with the LIGAND program of Munson and Rodbard (1980).

#### Measurements of the PKC*α* translocation

An acinar cell suspension was dissolved in 2 ml RPMI and added on top of 2 ml RPMI + 20% FCS. After 10 min on ice, the upper 2 ml layer was removed and the lower part was centrifuged and the pellet was resuspended in 6 ml HANK's buffer in a concentration of 3 × 10^6^ cells ml^−1^. Stimulation with methacholine or PMA was performed at 37°C. The reaction was stopped with 3 ml ice-cold buffer 1 (5 mM TrisHEPES, 50 mM mannitol, 0.25 mM MgCl_2_ pH=7.5). After centrifugation, the pellet was resuspended in 1 ml buffer 1 and cell lysis was induced by pottering the suspension (× 40, *d*=0.120 mm). The cells were fractionated by centrifugation, the first run was 12 400 **g**, 4°C, 20 min, the pellet contained whole cells, cell debris, nuclei and mitochondria. The supernatant was centrifuged at 200 000 **g**, 4°C for 1 h. The small pellet, which contained the membrane fraction, was resuspended in 30 *μ*l buffer 2 (20 mM Tris, pH=7.3, 2 mM EGTA, 3 *μ*g ml^−1^ aprotinin, 0.1 mM PMSF). The suspension was sonicated for 5 s and 5 *μ*l was used for Bradford assay to determine the protein concentration. In all, 10 *μ*g protein was used for Western blotting. After blocking overnight in 4% milk, the blot was incubated with the first anti-body PKC*α* (1 : 2500) for 1 h at room temperature and for another 1 h with secondary antibody rabbit (1 : 2000). Protein kinase C translocation was determined by comparing the membrane bound fraction with and without stimulation, both band were included. Since the muscarinic M_3_-receptor number related to membrane protein levels did not change after irradiation (see Results), protein levels of PKC*α* were also normalised initially against membrane protein levels, which were then routinely used for equal loading. This was confirmed with Na-K-ATPase as a loading control, using a first antibody Na-K-ATPase (1 : 15 000) for 1 h at room temperature and for another 1 h with secondary antibody goat (1 : 10 000).

### Statistical analysis

Paired analyses were performed using the Student *t*-test to evaluate statistical significance. Data values are expressed as mean±s.e. Statistical significance was defined as *P*-value of 0.05 or less.

## RESULTS

### Mobilisation of intracellular calcium

**Figure 1:**
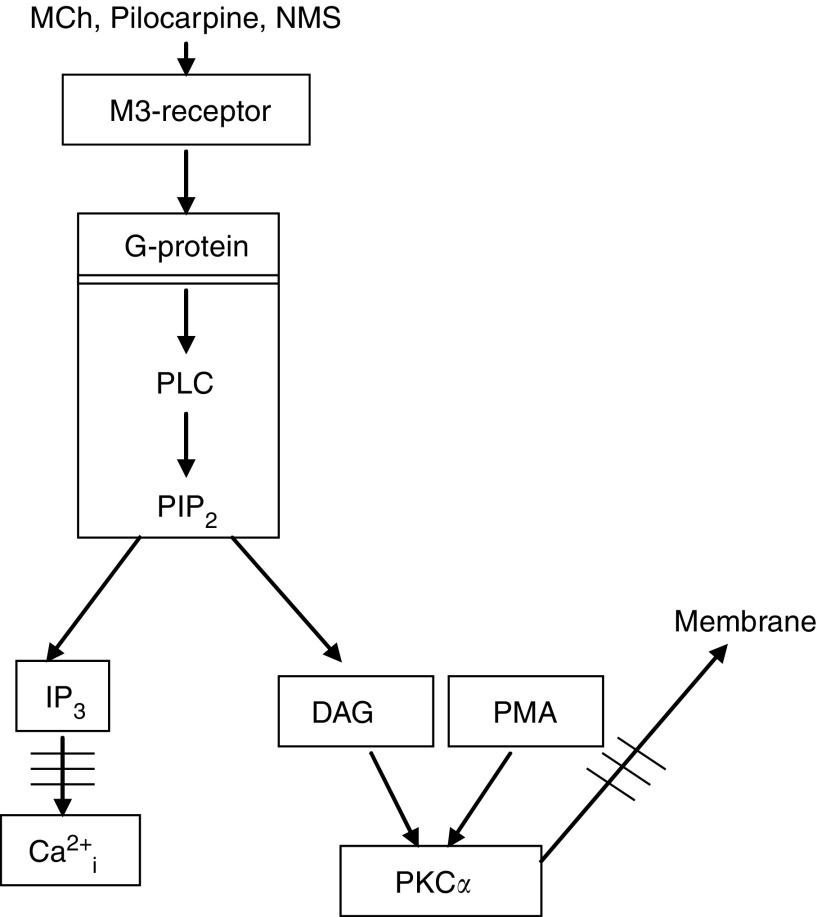
Schematic overview of muscarinic acetylcholine receptor signalling pathway.

**Figure 2:**
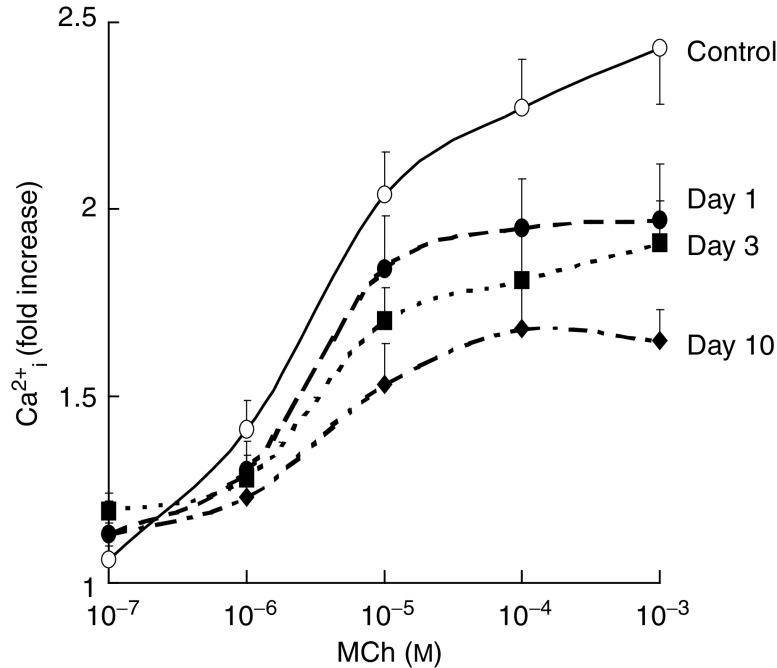
Effect of *in vivo* single dose (15 Gy) irradiation on the *ex vivo* metacholine-induced Ca^2+^_i_mobilisation. Ca^2+^_i_ was calculated from the fluorescence at two excitation wavelengths (340 and 380 nm), in Fura-2-A/M-loaded isolated rat parotid gland cells (1 × 10^6^ cells ml^−1^) in the presence of 1 mM Ca^2+^. Basal [Ca^2+^_i_]: 104±7 nM for unirradiated glands, and 130±10, 103±7, and 151±17 nM at 1, 3 and 10 days after irradiation, respectively. Each point represents five or more independent measurements. Significantly different from unirradiated situation: ^*^P<0.05 (Student's *t*-test).

As the increase in intracellular free calcium is the dominant mechanism that drives the watery secretion in parotid glands (Petersen, 1986), we first measured changes in muscarinic acetylcholine receptor-induced alterations in Ca^2+^_i_ mobilisation in isolated acinar cells (see Figure 1). After isolation, 95% of the cells were of the acinar cell type, as judged by their shape and content of vesicles. The *in vivo* irradiation did not change the number or type of cells per mg tissue isolated from the parotid glands (data not shown). Unirradiated parotid gland cells had a basal intracellular free calcium level [Ca^2+^_i_] of 104±7 nM, whereas 1, 3 and 10 days after irradiation the cells had a [Ca^2+^_i_] of 130±10, 103±7 and 151±17 nM, respectively. Figure 2 shows the relative increase in initial peak [Ca^2+^_i_] response as a function of the concentration of the specific muscarinic acetylcholine receptor agonist methacholine (MCh). Already at day 1 after 15 Gy of *in vivo* irradiation, the agonist-induced Ca^2+^_i_ mobilisation was significantly impaired. This response further declined with time after radiation, with acinar cells isolated 10 days after *in vivo* irradiation showing a 50–60% reduction in Ca^2+^_i_ mobilisation at 10^−3^ M MCh. These data suggest that the acute decrease in saliva secretion after radiation may be due to a reduced receptor-effector signaling.

### Receptor density and affinity

**Figure 3:**
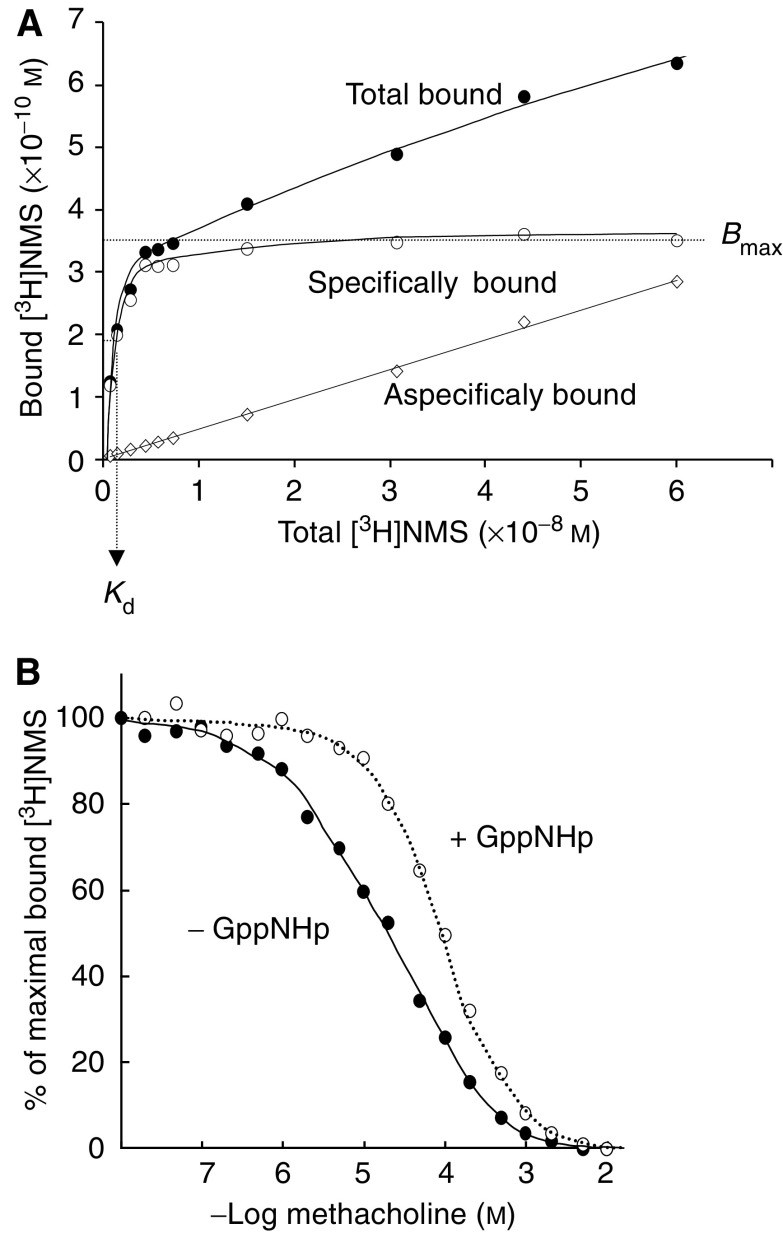
^3^H-NMS binding to isolated parotid gland membranes. (**A**) Typical example of total (•) and nonspecific ^3^H-NMS binding (◊) (^3^H-NMS binding in the presence of an excess amount of unlabelled ligand) to membranes from rat parotid glands allow the calculation of specific ^3^H-NMS binding (○). (**B**) Inhibition of ^3^H-NMS binding to rat parotid gland muscarinic receptors by the full muscarinic receptor agonist methacholine in the absence (•) or presence (○) of the stable GTP-analogue GppNHp. Lines are best computer-fit curves according to a two-site (solid line) or a one-site (dotted line) binding model. The two binding sites revealed in the absence of GppNHp are converted into a single site in the presence of GppNHp. Results from one, typical experiment.

To identify the radiation target for cellular dysfunction, we investigated various ‘players’ in the signal transduction pathway upstream of calcium mobilisation. The muscarinic acetylcholine M_3_-acetylcholine (M_3_) receptor is the main inducer of water secretion and of Ca^2+^_i_ mobilisation in the rat parotid gland (Nauntofte, 1992). The reduced secretory potential after X-irradiation may have resulted from radiation-induced reduction in the number of receptors, the affinity between the receptor and a ligand, or a reduced coupling of the receptor to its G-protein. To investigate this, the binding capacities and affinities of the parotid gland muscarinic acetylcholine receptors were examined. Hereto, receptor saturation binding experiments were performed on isolated membranes from *in vivo* irradiated parotid glands. The membranes were incubated with tritiated *N*-methyl-scopolamine (^3^H-NMS) as a muscarinic acetylcholine receptor radioligand. The incubation time used (30 min) was found to allow full equilibration of ligand binding (data not shown). Figure 3A is a typical example of a radioligand binding curve for parotid gland membranes. The nonspecific binding that was observed in the presence of excess cold ligand (unlabelled dexetimide) was substracted from the total amount of ^3^H-NMS bound protein, to yield the curve for specific binding. In membranes isolated from glands of non-irradiated animals, ^3^H-NMS bound to a single binding site (see methods), with a maximal binding capacity (*B*_max_) of 39±12 fmol mg^−1^ protein and a dissociation constant (p*K*_d_) of 9.9±0.11 nM. These parameters were not altered at any time after irradiation (Table 1), indicating that *in vivo* irradiation had not affected the binding capacities or affinities of muscarinic acetylcholine receptors in isolated membranes from the parotid glands. As the binding capacity is a measure for the number of receptors with specific affinity for the ligand, and the dissociation constant is a measure for the strength of the ligand binding, these observations indicate that the reduced saliva secretion/Ca^2+^_i_ mobilisation after irradiation is not caused by a reduced number of receptors or a reduced ligand-receptor-binding affinity.

Secondly, we investigated whether there was a change in parotid gland M_3_-receptor-G protein coupling efficiency. Hereto, isolated membranes from the parotid gland were incubated with 1 *μ*M of ^3^H-NMS to achieve maximal receptor binding. Subsequently, the ^3^H-NMS was replaced dose dependently by the full muscarinic acetylcholine agonist, MCh, as shown in Figure 3B (closed symbols). The displacement curve in membranes from unirradiated parotid glands, when measured in the absence of the GTP-analogue 5′-guanylylimido diphosphate (GppNHp), best fitted a two-site model (P<0.05), indicative for the existence of 66% high-affinity (p*K*_d_=5.4: Table 2)- and 34% low-affinity (p*K*_d_=4.4)-binding site for MCh. This is consistent with previously published data for the carbachol-induced displacement of ^3^H-NMS in parotid acinar cell membranes (Dai and Baum, 1993) and can be interpreted as showing the receptor–G-protein complex being the high-affinity state and the receptor in its G-protein-free state being low-affinity state (Wess *et al*, 1995). Further confirmation that this displacement curve indicates the existence of two separate binding sites with different affinity was obtained in experiments where the stable GppNHp was added in excess to dissociate all receptor–G-protein complexes (Figure 3, open symbols). In the presence of GppNHp, the displacement curve was shifted to the right and was best fitted assuming a one-site model (p*K*_d_=4.4: Table 2), indicating that the high-affinity site was indeed lost. As shown in Table 2, methacholine p*K*_d_ and *B*_max_ were unchanged after irradiation, irrespective of whether GppNHp was present or not. Also, radiation did not significantly alter the proportions of high- and low-affinity-binding sites. Thus, *in vivo* irradiation of parotid glands does not affect the number of muscarinic acetylcholine receptors, nor the binding of ligands to the receptor nor the binding of the receptor to its G-protein.

**Figure 4:**
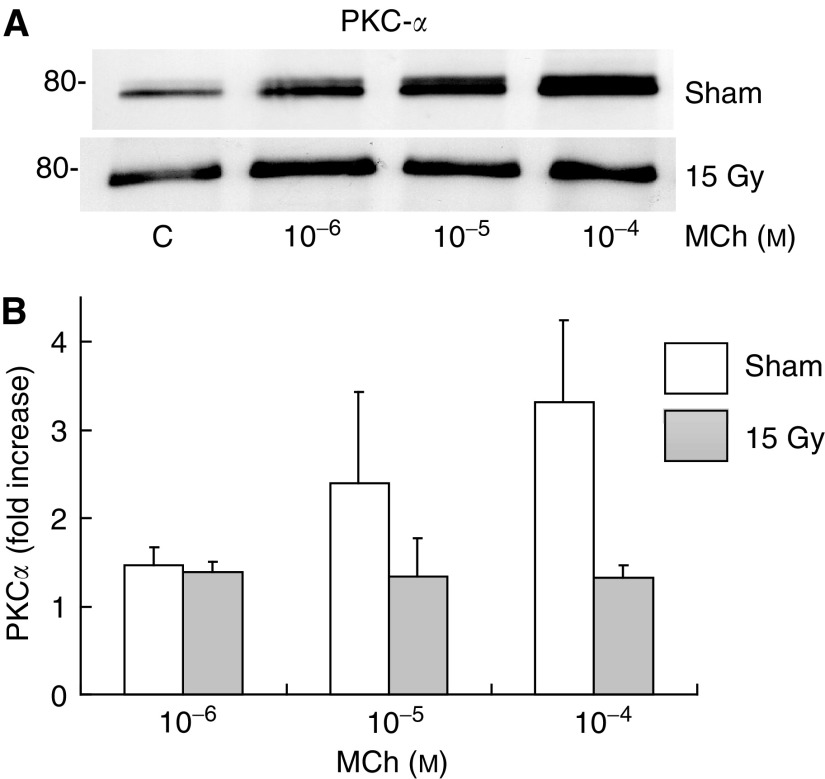
Changes in the levels of membrane-bound PKC*α* following methacholine stimulation of intact rat parotid gland acinar cells, 10 days after *in vivo* (sham-) irradiation. Isolated parotid gland cells were treated with methacholine for 2 min at the indicated doses. (**A**) Protein kinase C alpha expression was determined by immunoblot analysis as described under ‘Materials and Methods’. 80- indicates molecular mass marker. Always 10 *μ*g of protein used. (**B**) Diagram of at least three independent experiments. Significantly different from unirradiated situation: ^*^P<0.05 (Student's *t*-test).

The above implies that the damage induced by radiation and causing the impaired Ca-response (Figure 2) is localised downstream from the muscarinic acetylcholine-receptor–G-protein interaction. In an attempt to further pinpoint the exact localisation of the radiation-induced damage, the activation of PKC*α* was assessed. Protein kinase C alpha is activated by DAG, which is formed from Gq-protein-coupled activation of PLC hydrolysis of PIP_2_ in parallel with IP_3_ (which induced the calcium mobilisation from internal stores) (Baum *et al* 1993, Sawaki *et al*, 1993) (Figure 1). If PKC*α* activation would be found not to be affected by irradiation, this would imply that the relevant radiation-induced damage occurs downstream from the hydrolysis of PIP_2_. To test this we measured the methacholine-induced translocation of PKC*α* to the cell membrane in cells from irradiated and unirradiated glands, as this has been shown to correlate well with the activation PKC*α* (Terzian and Rubin 1993; Feng and Hannun 1998; Oancea and Meyer 1998; Tanimura *et al*, 2002). Stimulation of intact non-irradiated acinar cells from the parotid gland with 10^−4^ MCh revealed that a 2 min incubation time induced the highest level of PKC*α* translocation to the plasma membrane (data not shown). Figure 4 shows that MCh induced a dose-dependent increase of PKC*α* in the cell membrane fraction. However, 10 days after *in vivo* irradiation of the parotid glands, this increase was completely abrogated. So, the relevant radiation-induced damage must be upstream PIP_2_ hydrolysis. To investigate if PKC*α* translocation to the membrane is induced by irradiation alone, we analysed all control blots of this study, comparing unirradiated samples with irradiated samples. No differences were observed between unirradiated and irradiated controls (ratio: 1±0.09 *vs* 0.94±0.13, *n*=15).

**Figure 5:**
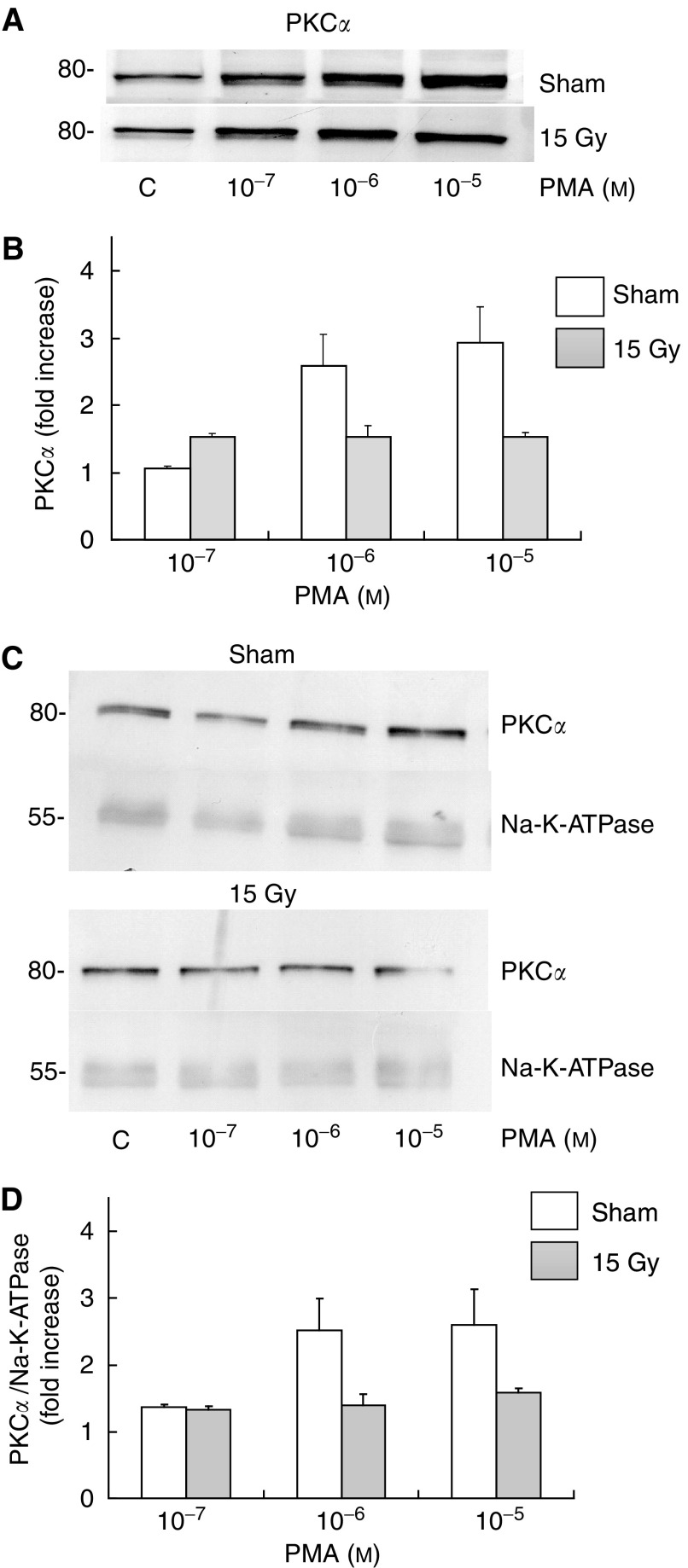
Changes in the levels of membrane-bound PKC*α* following PMA stimulation of intact rat parotid gland acinar cells, 10 days after *in vivo* (sham-) irradiation. Isolated parotid gland cells were treated with PMA for 3 min at the indicated doses. (**A**) Protein kinase C alpha expression was determined by immunoblot analysis as described under ‘Materials and Methods’. 80- indicate molecular mass marker. Always 10 *μ*g of protein was mounted (**B**) Diagram of at least three independent experiments. (**C**) Protein kinase C alpha and Na-K-ATPase expression was determined. (**D**) Diagram of at least three independent experiments. Significantly different from unirradiated situation: ^*^P<0.05 (Student's *t*-test).

To test whether the radiation-induced effect is due to an impairment of the membrane integrity in the anchoring of PKC*α* or due to primary damage to the G-protein-PIP_2_ hydrolysis process, we next tested whether radiation would also impair the ability of the phorbol ester PMA to cause PKC*α* membrane translocation. Maximal stimulation of PKC*α* translocation in intact nonirradiated acinar cells from the parotid gland was achieved with 3 min incubation with 1 *μ*M PMA (data not shown), and 3 min incubation time with PMA lead to a dose-dependent increase of PKC*α* in the cell membrane fraction (Figure 5A, B). Like for MCh, the activation of PKC*α* was completely abrogated in cells isolated 10 days after *in vivo* irradiation of the parotid glands, indicating that the (further) translocation of PKC*α* to the membrane is affected after *in vivo* irradiation irrespective of whether the stimulus is PLC-dependent or -independent.

To assure that radiation did not induce a global (specific) change in membrane proteins (without affecting total membrane proteins), we used Na-K-ATPase as a loading control for PMA-stimulated PKC translocation. The data shown in Figure 5C, D confirm that PMA-stimulated translocation of PKC*α* to the membrane is completely abrogated after irradiation without affecting total protein nor a specific membrane protein.

Together, our data show that radiation interferes with M_3_-muscarinic acetylcholine signalling pathway, which in the rat salivary gland may be the cause for the specific impairment in water, but not protein, secretion early after irradiation.

## DISCUSSION

Classically, the main target of radiation is believed to be the DNA, irradiation leading to mitotic death when cells attempt to divide or to apoptosis in nondividing cells. Progressive depopulation of the functional cell compartment then eventually leads to function loss of an organ. The slow turnover rate of salivary glands cells *vs* the rapid radiation-induced changes (see Introduction) is not compatible with mitotic cell death and failure in cell replacement. Therefore, it had been suggested (Stephens *et al*, 1989) that the acute damage to salivary gland was caused by apoptosis. These data, however, were based on essentially qualitative studies showing radiation-induced apoptosis in monkey salivary glands. Our more quantitative analysis in rat parotid glands did not detect apoptosis (Paardekooper *et al*, 1998) or cell loss (Coppes *et al*, 2000, 2001) to an extent that could explain the large drop in parotid gland function after irradiation. Therefore, the classical concept of DNA being the primary target was abandoned and, based on *in vivo* secretion studies, a defect in receptor-effector signal transduction of water secretion was proposed as a novel target, at least in rats (Coppes *et al*, 2000, 2001). The present observations of defective calcium mobilisation and PKC*α* translocation now directly demonstrate for the first time that radiation causes damage that interferes with intracellular receptor-mediated signalling, thereby compromising the function of an organ without inducing cell depletion.

Direct extrapolation of the data to the *in vivo* parotid gland function must be done with some precaution. Possibly, a defect in Ca mobilisation may have pronounced effects on the secretory capabilities of the gland, as the Ca^2+^ signalling is highly organised and compartmentalised (Berridge, 1996). In our case, only the initial peak of calcium release from intercellular stores was significantly affected indicating that a poor orchestration of the mobilisation of intracellular calcium through intra- and extracellular calcium channels could be responsible for the loss of function of the parotid acinar cells after *in vivo* irradiation, as Ca^2+^ release from the ER is responsible for the activation of basolaterally located K+ channels, whereas Ca^2+^ influx from the interstitium is responsible for the rise Ca^2+^ near the luminal membranes were the Cl^−^ channels are supposed to be located (Dissing *et al*, 1990). In conjunction, these two ions are responsible for the watery secretion (Petersen, 1986, 1992). Bodner *et al* (1984) did not observe changes in K^+^ efflux and uptake that follows intracellular Ca^2+^ levels. They, however, used epinephrine which stimulates *α*-adrenoceptors and does not stimulate water secretion as effectively as muscarinic receptors (Yu *et al*, 1987). In agreement with this we were not able to show changes in Ca^2+^ levels with the partial muscarinic agonist pilocarpine, indicating that the secretory process may be dampened and not completely abrogated. Radiation-induced salivary cellular dysfunction has been studied in the past. O'Connell *et al* (1999) could not find differences in the mobilisation of Ca^2+^ or other ion cotransport activity 12 months postirradiation of the submandibular gland. At this time, however, it is unlikely that the remaining acinar cells are the same as the ones that have been irradiated as the tissue cells have a lifespan of 60–120 days (Zajicek *et al*, 1989) and as such are not responsible for the acute irradiation damage.

It is not clear exactly how the Ca response is affected. The reduced Ca^2+^ mobilisation is not due to a reduction in number of muscarinic acetylcholine receptors, their affinity for the ligand or their coupling to G-protein. However, the translocation of PKC*α* is also affected after irradiation. The activation of PKC*α* is a complex process. After synthesis as a catalytically inactive protein it is converted into an active enzyme by three functional phosphorylations, whereafter it is released from the ribosomes to the cytosol (Liu and Heckman, 1998). Radiation had no effect on the total levels of cytosolic PKC*α* (data not shown), implying that it did not interfere with these initial steps of the PKC*α* activation process nor through altered gene expression. Next, DAG and Ca^2+^ synergise with *cis*-unsaturated fatty acids whereafter the C1 ligand acts as a hydrophobic anchor holding PKC*α* in the membrane. We found that PKC*α* binding to the membrane after DAG formation (via receptor stimulation) and after direct stimulation (by PMA) was reduced after irradiation, indicating that the process of translocation to the membrane seems to be affected. The cell membrane has been suggested to be a target for radiation (Konings and Drijver, 1979, Somosy, 2000). Membrane permeabilisation induced by saponin resulted in stable PKC*α* translocation to the membrane (Tanimura *et al*, 2002), furthermore it was suggested that PKC*α* may act as an amplifier of Ca^2+^ oscillation. Calcium drives the watery secretion by opening the basolateral K^+^ and apical Cl^−^ channels, and the osmotic gradient thus created leads to water movement into the lumen both through transcellular and paracellular processes. Paracellular water movement involves tight junction modulation (Hashimoto *et al*, 2000). Opening and closure of tight junctions is modulated by classical PKCs (Stuart and Nigam, 1995). Possibly, in conjunction with the affected calcium mobilisation, a disturbed PKC signalling may be involved in the irradiation-induced reduction in *in vivo* saliva secretion. In agreement with this, recently, Takagi *et al* (2003) showed a pertinent disorder of the water channel aquaporin 5 in acinar cells of irradiated submandibular glands, indicating possibly affection of paracellular pathways, which regretfully cannot be measured by the methods used in this study. Another possibility is that radiation-induced ceramide production (see Reynolds *et al*, 2004) and subsequent blocking of CRAC channels as has been shown in T lympocytes (Lepple-Wienhues *et al*, 1999) may play a role in the observed disturbance of cellular signalling.

This study demonstrates for the first time that the response of cells on agonist activation of a G-protein-coupled receptor-signal transduction pathway is affected after *in vivo* irradiation. Unraveling of the mechanism of early radiation-induced damage may reveal other ways to interfere with radiation-induced damage.

## Figures and Tables

**Table 1:** Receptor density (*B*_max_) and ^3^H-NMS-binding affinity (*K*_d_) of muscarinic receptors in isolated parotid gland membranes after *in vivo* irradiation with 15 Gy X-rays

**Time (days) after 15 Gy**	**p*K*_d_ −log (M)**	***B*_max_ (fmol/mg)**	***n***
Control	9.9±0.1	39±12	6
1	9.7±0.1	38±15	5
3	9.8±0.1	51±9	4
10	9.8±0.1	42±13	5

**Table 2:** Replacement of ^3^H-NMS binding by the full muscarinic receptor agonist methacholine in the absence and presence of GppNHp in isolated parotid gland membranes after *in vivo* irradiation with 15 Gy X-rays

**Time (days) after 15 Gy**	**GppNHp**	**p*K*_d_ −log (M)**	**Proportion of *B*_max_(%)**
Control	−	5.4±0.1	66±6
		4.4±0.1	34±6
	+	4.7±0.1	100
			
1	−	5.5±0.5	57±26
		4.2±0.4	43±26
	+	4.6±0.1	100
			
3	−	5.6±0.3	54±14
		4.4±0.3	46±16
	+	4.6±0.2	100
			
10	−	5.4±0.3	69±21
		4.2±0.1	31±21
	+	4.7±0.1	100

*B*_max_ represents the maximal binding per binding site, and p*K*_d_ represents the affinity of binding per binding site. Each value is the average of at least five independent measurements (membranes obtained from separate animals).

## References

[bib1] Baum BJ, Dai Y, Hiramatsu Y, Horn VJ, Ambudkar IS (1993) Signaling mechanisms that regulate saliva formation. Crit Rev Oral Biol Med 4: 379–384 8104047 10.1177/10454411930040031701

[bib2] Berridge MJ (1996) Microdomains and elemental events in calcium signalling. Cell Calcium 20: 95–96 8889200 10.1016/s0143-4160(96)90098-6

[bib3] Bodner L, Kuyatt BL, Hand AR, Baum BJ (1984) Rat parotid cell function *in vitro* following X irradiation *in vivo*. Radiat Res 97(2): 386–395 6198673

[bib4] Burlage FR, Coppes RP, Meertens H, Stokman MA, Vissink A (2001) Parotid and submandibular/sublingual salivary flow during high dose radiotherapy. Radiother Oncol 61: 271–274, doi:10.1016/S0167-8140(01)00427-3 11730996 10.1016/s0167-8140(01)00427-3

[bib5] Coppes RP, Roffel AF, Zeilstra LJ, Vissink A, Konings AW (2000) Early radiation effects on muscarinic receptor-induced secretory responsiveness of the parotid gland in the freely moving rat. Radiat Res 153: 339–346, doi: 10.1043/0033-7587(2000)153<0339:EREOMR>2.0.CO;2 10669557 10.1667/0033-7587(2000)153[0339:ereomr]2.0.co;2

[bib6] Coppes RP, Vissink A, Zeilstra LJ, Konings AW (1997a) Muscarinic receptor stimulation increases tolerance of rat salivary gland function to radiation damage. Int J Radiat Biol 72: 615–625, doi:10.1080/095530097143112 9374441 10.1080/095530097143112

[bib7] Coppes RP, Zeilstra LJ, Kampinga HH, Konings AW (2001) Early to late sparing of radiation damage to the parotid gland by adrenergic and muscarinic receptor agonists. Br J Cancer 85: 1055–1063, doi:10.1054/bjoc.2001.2038 11592779 10.1054/bjoc.2001.2038 PMC2375094

[bib8] Coppes RP, Zeilstra LJ, Vissink A, Konings AW (1997b) Sialogogue-related radioprotection of salivary gland function: the degranulation concept revisited. Radiat Res 148: 240–247 9291355

[bib9] Dai Y, Baum BJ (1993) Relationship between muscarinic receptor occupancy and response in rat parotid acinar cells. Am J Physiol 265: G1122–G1127 8279563 10.1152/ajpgi.1993.265.6.G1122

[bib10] Dai YS, Ambudkar IS, Horn VJ, Yeh CK, Kousvelari EE, Wall SJ, Li M, Yasuda RP, Wolfe BB, Baum BJ (1991) Evidence that M3 muscarinic receptors in rat parotid gland couple to two second messenger systems. Am J Physiol 261: C1063–C1073 1722644 10.1152/ajpcell.1991.261.6.C1063

[bib11] Dissing S, Nauntofte B, Sten-Knudsen O (1990) Spatial distribution of intracellular, free Ca^2+^ in isolated rat parotid acini. Pflugers Arch 417: 1–12 2293199 10.1007/BF00370762

[bib12] Feng X, Hannun YA (1998) An essential role for autophosphorylation in the dissociation of activated protein kinase C from the plasma membrane. J Biol Chem 273: 26870–26874 9756933 10.1074/jbc.273.41.26870

[bib13] Grynkiewicz G, Poenie M, Tsien RY (1985) A new generation of Ca^2+^ indicators with greatly improved fluorescence properties. J Biol Chem 260: 3440–3450 3838314

[bib14] Hashimoto S, Ochiai S, Muramatsu T, Shimono M (2000) Tight junctions in the rat parotid gland. Eur J Morphol 38: 263–267 10980678 10.1076/0924-3860(200010)38:4;1-o;ft263

[bib15] Konings AW, Drijver EB (1979) Radiation effects on membranes. I. Vitamin E deficiency and lipid peroxidation. Radiat Res 80: 494–501 515350

[bib16] Lepple-Wienhues A, Belka C, Laun T, Jekle A, Walter B, Wieland U, Welz M, Heil L, Kun J, Busch G, Weller M, Bamberg M, Gulbins E, Lang F (1999) Stimulation of CD95 (Fas) blocks T lymphocyte calcium channels through sphingomyelinase and sphingolipids. Proc Natl Acad Sci USA 96: 13795–13800 10570152 10.1073/pnas.96.24.13795 PMC24144

[bib17] Liu WS, Heckman CA (1998) The sevenfold way of PKC regulation. Cell Signal 10: 529–542, doi:10.1016/S0898-6568(98)00012-6 9794251 10.1016/s0898-6568(98)00012-6

[bib18] Lowry OH, Rosebrough NJ, Farr AL, Randall RJ (1951) Protein measurement with folin phenol reagent. J Biol Chem 193: 265–276 14907713

[bib19] Melvin JE (1999) Chloride channels and salivary gland function. Crit Rev Oral Biol Med 10: 199–209 10759422 10.1177/10454411990100020601

[bib20] Munson PJ, Rodbard D (1980) Ligand: a versatile computerized approach for characterization of ligand-binding systems. Anal Biochem 107: 220–239 6254391 10.1016/0003-2697(80)90515-1

[bib21] Nauntofte B (1992) Regulation of electrolyte and fluid secretion in salivary acinar cells. Am J Physiol 263: G823–G837 1476190 10.1152/ajpgi.1992.263.6.G823

[bib22] Nishizuka Y (1992) Intracellular signaling by hydrolysis of phospholipids and activation of protein kinase C. Science 258: 607–614 1411571 10.1126/science.1411571

[bib23] O'Connell AC, Redman RS, Evans RL, Ambudkar IS (1999) Radiation-induced progressive decrease in fluid secretion in rat submandibular glands is related to decreased acinar volume and not impaired calcium signaling. Radiat Res 151: 150–158 9952299

[bib24] Oancea E, Meyer T (1998) Protein kinase C as a molecular machine for decoding calcium and diacylglycerol signals. Cell 95: 307–318 9814702 10.1016/s0092-8674(00)81763-8

[bib25] Oliver C (1980) Isolation and maintenance of differentiated exocrine gland acinar cells *in vitro*. *In Vitro* 16: 297–305 7399544 10.1007/BF02618335

[bib26] Paardekooper GM, Cammelli S, Zeilstra LJ, Coppes RP, Konings AW (1998) Radiation-induced apoptosis in relation to acute impairment of rat salivary gland function. Int J Radiat Biol 73: 641–648, doi: 10.1080/095530098141898 9690682 10.1080/095530098141898

[bib27] Peter B, Van Waarde MA, Vissink A, 's-Gravenmade EJ, Konings AW (1994) Radiation-induced cell proliferation in the parotid and submandibular glands of the rat. Radiat Res 140: 257–265 7938475

[bib28] Petersen OH (1986) Calcium-activated potassium channels and fluid secretion by exocrine glands. Am J Physiol 251: G1–G13 2425634 10.1152/ajpgi.1986.251.1.G1

[bib29] Petersen OH (1992) Stimulus-secretion coupling: cytoplasmic calcium signals and the control of ion channels in exocrine acinar cells. J Physiol 448: 1–51 1375633 10.1113/jphysiol.1992.sp019028 PMC1176186

[bib30] Reynolds CP, Maurer BJ, Kolesnick RN (2004) Ceramide synthesis and metabolism as a target for cancer therapy. Cancer Lett 206: 169–180 15013522 10.1016/j.canlet.2003.08.034

[bib31] Sawaki K, Hiramatsu Y, Baum BJ, Ambudkar IS (1993) Involvement of G alpha q/11 in m3-muscarinic receptor stimulation of phosphatidylinositol 4,5 bisphosphate-specific phospholipase C in rat parotid gland membranes. Arch Biochem Biophys 305: 546–550, doi:10.1006/abbi.1993.1459 8396894 10.1006/abbi.1993.1459

[bib32] Somosy Z (2000) Radiation response of cell organelles. Micron 31: 165–181, doi:10.1016/S0968-4328(99)00083-9 10588063 10.1016/s0968-4328(99)00083-9

[bib33] Stephens LC, King GK, Peters LJ, Ang KK, Schultheiss TE, Jardine JH (1986) Acute and late radiation injury in rhesus monkey parotid glands. Evidence of interphase cell death. Am J Pathol 124: 469–478 3766705 PMC1888342

[bib34] Stephens LC, Schultheiss TE, Small SM, Ang KK, Peters LJ (1989) Response of parotid gland organ culture to radiation. Radiat Res 120: 140–153 2798777

[bib35] Stuart RO, Nigam SK (1995) Regulated assembly of tight junctions by protein kinase C. Proc Natl Acad Sci USA 92: 6072–6076 7597083 10.1073/pnas.92.13.6072 PMC41644

[bib36] Takagi K, Yamaguchi K, Sakurai T, Asari T, Hashimoto K, Terakawa S (2003) Secretion of saliva in X-irradiated rat submandibular glands. Radiat Res 159: 351–360, doi: 10.1043/0033-7587(2003)159<0351:SOSIXI>2.0.CO;2 12600238 10.1667/0033-7587(2003)159[0351:sosixi]2.0.co;2

[bib37] Takeda I, Kizu Y, Yoshitaka O, Saito I, Yamane GY (2003) Possible role of nitric oxide in radiation-induced salivary gland dysfunction. Radiat Res 159: 465–470, doi: 10.1043/0033-7587(2003)159<0465:PRONOI>2.0.CO;2 12643791 10.1667/0033-7587(2003)159[0465:pronoi]2.0.co;2

[bib38] Tanimura A, Nezu A, Morita T, Hashimoto N, Tojyo Y (2002) Interplay between calcium, diacylglycerol, and phosphorylation in the spatial and temporal regulation of PKCalpha-GFP. J Biol Chem 277: 29054–29062, doi:10.1074/jbc.M201130200 11997388 10.1074/jbc.M201130200

[bib39] Taylor SE, Miller EG (1999) Preemptive pharmacologic intervention in radiation-induced salivary dysfunction. Proc Soc Exp Biol Med 221: 14–26 10320627 10.1046/j.1525-1373.1999.d01-48.x

[bib40] Terzian AR, Rubin RP (1993) Translocation of the alpha-isozyme of protein kinase C during stimulation of rat parotid acinar cells by phorbol ester and carbachol. Arch Oral Biol 38: 1051–1056 8141666 10.1016/0003-9969(93)90166-j

[bib41] Tojyo Y, Matsui S, Tanimura A, Matsumoto Y (1992) Relationship between cytosolic Ca^2+^ concentration and amylase release in rat parotid acinar cells following muscarinic stimulation. Biochim Biophys Acta 1134: 278–284 1373078 10.1016/0167-4889(92)90187-g

[bib42] Vissink A, Down JD, Konings AW (1992) Contrasting dose-rate effects of gamma-irradiation on rat salivary gland function. Int J Radiat Biol 61: 275–282 1351916 10.1080/09553009214550911

[bib43] Wess J, Blin N, Mutschler E, Bluml K (1995) Muscarinic acetylcholine receptors: structural basis of ligand binding and G protein coupling. Life Sci 56: 915–922 10188793 10.1016/0024-3205(95)00028-5

[bib44] Yu JH, Mark MR, Redman RS (1987) Effect of clonidine on secretion of fluid and ions by the parotid and submandibular glands of the rat. Arch Oral Biol 32: 27–33 3479071 10.1016/0003-9969(87)90150-6

[bib45] Zajicek G, Schwartz-Arad D, Arber N, Michaeli Y (1989) The streaming of the submandibular gland. II: Parenchyma and stroma advance at the same velocity. Cell Tissue Kinet 22: 343–348 2611851 10.1111/j.1365-2184.1989.tb00219.x

